# Mouse Papillomavirus L1 and L2 Are Dispensable for Viral Infection and Persistence at Both Cutaneous and Mucosal Tissues

**DOI:** 10.3390/v13091824

**Published:** 2021-09-14

**Authors:** Sarah Brendle, Jingwei J. Li, Nancy M. Cladel, Debra A. Shearer, Lynn R. Budgeon, Karla K. Balogh, Hannah Atkins, Marina Costa-Fujishima, Paul Lopez, Neil D. Christensen, John Doorbar, Thomas T. Murooka, Jiafen Hu

**Affiliations:** 1 The Jake Gittlen Laboratories for Cancer Research, Hershey, PA 17033, USA; ganzelly@gmail.com (S.B.); jenny.jingwei.li@gmail.com (J.J.L.); ncladel@gmail.com (N.M.C.); dshearer@pennstatehealth.psu.edu (D.A.S.); lynn.budgeon@comcast.net (L.R.B.); kkb2@psu.edu (K.K.B.); ndc1@psu.edu (N.D.C.); 2 Department of Pathology, Pennsylvania State University College of Medicine, Hershey, PA 17033, USA; 3 Laboratory Medicine, Department of Pathology, Division of Comparative Medicine, University of North Carolina, Chapel Hill, NC 27599, USA; hannah_atkins@med.unc.edu; 4 Department of Immunology, University of Manitoba, Winnipeg, MB R3E 0T5, Canada; costafum@myumanitoba.ca (M.C.-F.); Paul.Lopez@umanitoba.ca (P.L.); thomas.murooka@umanitoba.ca (T.T.M.); 5 Department of Microbiology and Immunology, Pennsylvania State University College of Medicine, Hershey, PA 17033, USA; 6 Department of Pathology, Division of Virology, University of Cambridge, Tennis Court Road, Cambridge CB21 QP, UK; jd121@cam.ac.uk

**Keywords:** mouse papillomavirus, L1 mutant, L2 mutant, viral production, tumor growth, RCA, ISH, IHC, RNAscope, TEM, viral copy number, qPCR

## Abstract

Papillomavirus L1 and L2, the major and minor capsid proteins, play significant roles in viral assembly, entry, and propagation. In the current study, we investigate the impact of L1 and L2 on viral life cycle and tumor growth with a newly established mouse papillomavirus (MmuPV1) infection model. MmuPV1 L1 knockout, L2 knockout, and L1 plus L2 knockout mutant genomes (designated as L1ATGko-4m, L2ATGko, and L1-L2ATGko respectively) were generated. The mutants were examined for their ability to generate lesions in athymic nude mice. Viral activities were examined by qPCR, immunohistochemistry (IHC), in situ hybridization (ISH), and transmission electron microscopy (TEM) analyses. We demonstrated that viral DNA replication and tumor growth occurred at both cutaneous and mucosal sites infected with each of the mutants. Infections involving L1ATGko-4m, L2ATGko, and L1-L2ATGko mutant genomes generally resulted in smaller tumor sizes compared to infection with the wild type. The L1 protein was absent in L1ATGko-4m and L1-L2ATGko mutant-treated tissues, even though viral transcripts and E4 protein expression were robust. Therefore, L1 is not essential for MmuPV1-induced tumor growth, and this finding parallels our previous observations in the rabbit papillomavirus model. Very few viral particles were detected in L2ATGko mutant-infected tissues. Interestingly, the localization of L1 in lesions induced by L2ATGko was primarily cytoplasmic rather than nuclear. The findings support the hypothesis that the L2 gene influences the expression, location, transport, and assembly of the L1 protein in vivo.

## 1. Introduction

L1 and L2, the papillomavirus major and minor capsid proteins, have been studied extensively in vitro [1,2]. As a result of the species-specificity of papillomaviruses, no laboratory animals support human papillomavirus (HPV) infection [3]. The cottontail rabbit papillomavirus (CRPV) model was the first in vivo model to test the function of L1 [4,5]. Our group and others have shown that neither L1 nor L2 proteins are required for tumor outgrowth in the CRPV/rabbit model [4,6,7]. These proteins can be eliminated by either mutating the open reading frame ATG to ACG, as we did for our mutants, or by deleting a portion of the genes as has been done by others [4,8,9]. We found that the CRPV L1ATGko mutant genome induced larger tumors than wild-type virus [8]. However, the deletion of sequences located in the 3′ half of the CRPV L2 and the entire L1 yielded a genome, which failed to induce tumors, indicating that at least a portion of this region is required for productive CRPV infections [4,9]. The CRPV model is a surrogate model for high-risk HPV infection [10]. However, the laboratory domestic rabbit is not a natural host for CRPV infection, and only very low amounts of virus can be detected in the infected tissues, indicating that the viral life cycle may be compromised to some extent [7]. Thus, the rabbit model has limited our ability to fully evaluate the in vivo roles of both L1 and L2 in the viral life cycle.

The papillomavirus community has gained significant understanding of L1 and L2 at the molecular level [1,2], but the functions of L1 and L2 in viral life cycle under in vivo conditions are still poorly understood [1]. Using a raft culture system to mimic in vivo viral production, investigators studied the HPV31 life cycle and revealed some unique functions of L2 [11]. Yet, this system is unable to capture the full complexity of host involvement during natural viral infections [12].

**Figure 1:**
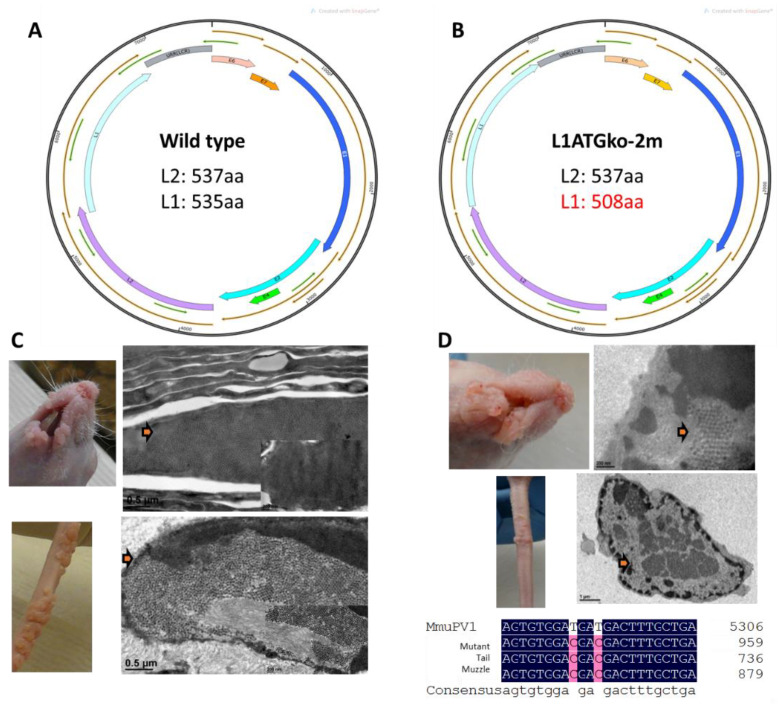
L1 and viral particles are detected at muzzle and tail sites in two animals with tumors induced by the L1ATGko-2m mutant. L1 with the first two methionines mutated to ACG (L1ATGko-2m) results in a shorter L1 (508aa) if the protein is expressed from the third methionine of the sequence (**B**), minus 27aa of the wild-type L1 (**A**). Viral particles (**C** and **D**, arrows) were detected in wild-type (**C**) and L1ATGko-2m mutant (**D**) induced muzzle lesions (upper panel) and in tail lesions (lower panel) by transmission electron microscopy (TEM). Mutation of the first two L1 methionines was confirmed in the viral DNA extracted from these lesions by DNA sequencing following rolling circling amplification (bottom, **D**). L1 was also detected in these lesions by immunohistochemistry (IHC) with an in-house monoclonal antibody (MPV. B9) against the mouse papillomavirus major capsid protein L1 (data not shown).

The mouse papillomavirus (MmuPV1) was first identified in an athymic nude mouse colony in 2011 [13]. Since then, MmuPV1 has been shown to infect both cutaneous and mucosal sites in mice [14,15,16,17,18]. Abundant virions are produced, indicating that the complete viral life cycle occurs in these infected tissues [14,16,19]. L1 and L2 of MmuPV1 share similar sequence homology with other human and animal papillomaviruses [20]. Theoretically, the MmuPV1 L1 protein can be encoded from any of the four N-terminal methionines (at the 1st, 2nd, 28th, and 30th amino acid residue, respectively) [21]. The third methionine at the 28th position incorporates the consensus motif (MxxWx7YLPP) for most L1s. An in vitro study demonstrated that MmuPV1 L1 proteins initiating from the 2nd methionine failed to form proper VLPs, but L1s starting from the 3rd and 4th methionine generated correctly formed VLPs in abundant quantities [21]. To further explore the role of L1 in the viral life cycle using this novel mouse model, we generated two L1 mutants. The first mutant, with the first two methionines abrogated (designated L1ATGko-2m) (Figure 1), would result in a protein of 508aa instead of 537aa if the protein were expressed from the next available (3rd) methionine. The second mutant, with all four methionines mutated (designated as LlATGko-4m), would result in a truncated protein of 362aa if the protein was expressed from the next available methionine.

The functions of MmuPV1 L2 and the interaction between L1 and L2 have not been studied previously [14]. Therefore, we generated two mutants: (1) A genome with the ATG of L2 mutated to ACG (designated L2ATGko) and (2) A double mutant with all four ATGs of L1 and the ATG of L2 mutated (designated L1-L2ATGko). These two mutants were used to explore the function of L1 and L2 individually and together in natural infections in the mouse.

The mutant genomes were encapsidated in HPV31 L1/L2 capsids to generate quasiviruses (QVs) using a previously established in vitro production system [22]. Outbred nude (Hsd:Nu) mice were challenged at cutaneous (tail and muzzle) and mucosal (vaginal, anal and oral) sites with the different QVs. The infections were tracked longitudinally for up to 22 weeks post-infection by employing our qPCR analysis [23]. The infected tissues were further examined for viral activity using virological and immunological assays developed in our laboratory [17,18]. Our results have demonstrated that neither L1 nor L2 protein is required for tumor growth at both cutaneous and mucosal tissues. Overall, infection with the mutants showed similar tissue tropism as infection with the wild-type virus, although there were variations in tissue susceptibility. L1 protein was detected in the nucleus in lesions induced by the wild-type but predominantly in the cytoplasm in lesions induced by the L2 mutant. No L1 signals were detected in lesions induced by the L1ATGko-4m and L1-L2ATGko mutants. Our findings agree with our previous studies in the CRPV/rabbit model that neither L1 nor L2 is essential for tumor growth and a recent report for MmuPV1 [8,24]. Our recent study also demonstrated that papillomavirus virions and viral DNA can initiate infections via the blood [25]. We postulate that human papillomavirus with mutated L1 or L2 resulting in dysfuncational capsid proteins may be still infectious and produce disease.

## 2. Materials and Methods

### 2.1. Mutant Genome Generation

L1 mutants were generated by mutating either the first two methionines (designated as L1ATGko-2m) or all four start codons (designated L1ATGko-4m) from ATG to ACG [21]. The L2 mutant was generated by mutating the ATG start codon to ACG (designated L2ATGko). The L1 plus L2 double mutant contained ATG to ACG mutations of all four potential start codons of L1 as well as the single start codon of L2 (designated L1-L2ATGko). The mutant genes were verified by DNA sequencing. Then, the BstEII/4107bp-RsrII/5491bp fragment containing the L1 mutations and/or the BsrGI/3007bp-BstEII/4107bp fragment containing the L2 mutation were ligated back into the wild-type genome backbone and sequenced to ensure that no additional mutations had been introduced during the amplification reactions.

Each mutant genome was released from the vector with BamHI digestion and retrieved with a gel extraction kit (QIAGEN, Germantown, MD, USA) to be used for quasivirus (QV) production as previously described [22]. HPV31 L1/L2 capsids were used to package the mutant genomes as the HPV31 L1/L2 capsid was shown to be highly effective for DNA delivery (our unpublished observations). QVs were freed of any externally attached DNA with Benzonase (Sigma-Aldrich, Inc., St. Louis, MO, USA) treatment and then purified by Opti-prep ultracentrifugation and examined by ELISA to evaluate capsid proteins. The fractions were harvested and tested for L1 and/or L2 presence with our in-house antibodies [26,27]. The fractions containing higher levels of L1 and/or L2 were stored to be used for infections.

### 2.2. Animals and Viral Infection

All mouse work was approved by the Institutional Animal Care and Use Committee of Pennsylvania State University’s College of Medicine and University of Manitoba. Female HSD outbred Foxn1^nu^/^nu^ nude mice (4–5 weeks old) were obtained from ENVIGO and were housed in sterile cages within sterile filter hoods and were fed sterilized food and water in the BL2 animal core facility. Female NU/J heterozygous mice were purchased from the Jackson’s laboratory and maintained in an animal core facility at the University of Manitoba. Mice were sedated i.p. with 0.1 mL/10 g body weight ketamine/xylazine mixture (100 mg/10 mg in10 mls ddH_2_O). Each nude mouse was infected with one mutant virus at both cutaneous and mucosal sites simultaneously. All animals were treated with Depo-Provera three days before scarification as described previously [18,23]. Muzzle and tail were abraded with a scalpel blade one day before viral infection. Some bleeding occurred, but care was taken to minimize the bleeding. For mucosal sites, the vaginal and anal tract were wounded by a Doctor’s Brushpick^TM^, and the base of the tongue was wounded with a microneedle as described previously [18,25]. Following scarification, animals were allowed to recover overnight. Infection in mice has been shown to be most efficient at day one post-pre-scarification, and our studies for infection all followed this protocol [19,22]. The following day, each animal was again anesthetized, and 10 µL of QVs (1.5 × 10^8^ genome DNA equivalent) were applied to each wounded site followed either by scratching with a TB needle for cutaneous sites or a Doctor’s Brushpick^TM^ at the mucosal sites [23,27]. To block the potential contamination by wild-type MmuPV1, we also used a neutralizing antibody MPV.A4 at the time of mutant virus infection, as described previously [25]. Animals were monitored weekly for tumor growth at the cutaneous sites, and photos were recorded. Infection at the mucosal sites were monitored by analysis of periodical lavages/swabs as previously reported [23].

### 2.3. Detection of Viral DNA by qPCR

DNA was extracted from lavage/swab specimens using the DNeasy kit (QIAGEN, Germantown, MD 20874, USA). Linearized MmuPV1 genome DNA was used for standard curve determination by Probe qPCR analysis (Brilliant III Ultra-Fast a probe PCR Master Mix, Agilent, Santa Clara, CA, USA). The primer pairs (5′GGTTGCGTCGGAGAACATATAA3′ and 5′CTAAAGCTAACCTGCCACATATC3′) and the probe 6-5′ FAM-TGCCCTTTCA/ZEN/GTGGGTTGAGGACAG 3′-IBFQ 3′) that amplifies a portion of E2 were used [25]. Oral, vaginal, and anal lavage samples were collected from mucosal sites, and DNA was extracted from these for qPCR analysis [23]. Viral copy numbers in 2 μL of the 50 μL DNA extracts were converted into equivalent DNA load using the formula 1ng viral DNA = 1.2 × 10^8^ copy numbers, (http://cels.uri.edu/gsc/cndna.html, accessed on 1 May 2015). The qPCR reactions were run in an AriaMx cycler (Agilent). Each reaction consisted of 500 nM specific primer pairs, 250 nM double-labeled probe. The following cycling conditions were applied: one cycle of 95 °C for 10 min followed by 40 cycles of 94 °C for 15 s and 60 °C for 1 min [17]. All samples were tested in at least duplicates. Viral titers were calculated according to the standard curve. In some cases, we also calculated the difference in cycle time (Ct) between the 18sRNA gene and viral DNA (ΔCt) [23]. Fold change (2^ΔCt^) demonstrated the relative viral DNA load in each sample as previously described [18].

### 2.4. Confirmation of Viral DNA by Rolling Circle Amplification and Sequencing

Our unpublished observations have shown that MmuPV1 does not tolerate foreign insertions such as GFP and luciferase. Such genomes reverted to wild type in vivo. To confirm whether infection was initiated and sustained by MmuPV1 L1 or L2ATGko, we amplified the viral genome directly from the DNA extract of lesions or lavages/biopsies using the illustraTempliPhi Amplification Kit (GE Healthcare life sciences, Piscataway, NJ, USA). A MmuPV1 specific primer mix covering the entire MmuPV1 genome was used for amplification (5′ ATG GAA ATC GGC AAA GGC 3′, 5′ ATG CAG GGC CCA TTA CCA3′, 5′ GGACTGTGAATTATAAAC 3′, 5′ GCCCGAAGACAACACCG CCACG3′, 5′CCTCCGCCTCGTCCC CAAATGG 3′, 5′ CCTCTGCGTCTTCTAGACG AGTTG 3′, 5′ GGTGTGGATGAGTTGTTAATACTC 3′, 5′ GCTACCTCCGCTACTAATT ACC 3′, 5′ GCCTTTGAGCCTGGGAGC 3′, 5′ GGGGATAAG ATGTTCTTC 3′). The amplified DNA was subsequently sequenced and aligned with the wild-type MmuPV1 using DNAMAN software.

### 2.5. Detection of Virus by ELISA, In Situ Hybridization, Immunohistochemistry, and Transmission Electron Microscope

Infected tissues were harvested at the termination of the experiments and stored in liquid nitrogen before being used for DNA and protein extraction. For ELISA, infected tissue suspension (2 µg protein/well) was used as an antigen for viral detection, as described previously [7]. A panel of anti-MmuPV1 L1 monoclonal antibodies generated in house (MPV.D8, J28, E12, B9, A4) were used for ELISA. Biopsies and tissues were fixed in 10% buffered formalin and embedded in paraffin. Sequential sections were cut for immunohistochemistry (IHC), in situ hybridization (ISH), RNA-ISH, and hematoxylin and eosin (H&E) analyses [17,25,28]. A 3913 bp EcoRV/BamH1sub genomic fragment of MmuPV1 was used as an in situ hybridization probe for the detection of MmuPV1 DNA in infected tissues [29]. The probe was biotinylated using the random priming method and diluted in a hybridization cocktail described in previous work. Access to target DNA was facilitated by incubation with 0.2 mg/mL pepsin in 0.1 N HCl at 37 °C for 8 min. After thorough washing, the biotinylated probe was applied and heated to 95 °C for 5 min to achieve the dissociation of target and probe DNA. Re-annealing was allowed to occur for 2 h at 37 °C. Target-bound biotin was detected using a streptavidin AP conjugate followed by colorimetric development in BCIP/NBT. Nuclear fast red (American Mastertech Inc., Lodi, CA, USA) was used as a counterstain for ISH. For L1 capsid detection by IHC, an in-house monoclonal antibody against denatured full-length MmuPV1 L1 (MPV.B9) and polyclonal antibody against group-specific antigen (GSA, Dako, Via Real Carpinteria, CA, USA) were used [17]. Detection was achieved using the ImmPRESS anti-mouse IgG polymer system (Vector #MP-7405) [29]. For MmuPV1 E4 detection, a rabbit anti-MmuPV1 E4 polyclonal antibody was used. Hematoxylin (Themo Scientific, Inc., Waltham, MA USA, USA) was used as the counterstain for IHC.

For transmission electron microscopy (TEM), the tissue was immersion-fixed for 24 h in Karnovsky’s fixative (5% glutaraldehyde/4% paraformaldehyde) in 0.1 M sodium cacodylate buffer, pH 7.3 [23]. Following fixation, the tissue was washed in 0.1 M sodium cacodylate buffer, post fixed in buffered 1% osmium tetroxide/1.5% potassium ferrocyanide, washed again with buffer, dehydrated in a graded series of ethanol, transferred to propylene oxide, and embedded in Spurr low viscosity resin. A total of 70–90 nm sections were cut with a diamond knife, mounted on copper grids, and stained with 2% aqueous uranyl acetate and lead citrate. The sections were examined in a JEOL JEM 1400 electron microscope, and images were recorded.

### 2.6. Western Blot Analysis for Detection of L1 Protein

Frozen tail and vagina tissues were crushed with a hammer while in liquid nitrogen and transferred to immunoprecipitation (IP) buffer (20 mM Tris HCL, pH 8, 137 mM NaCl, 1% Nonidet P-40, 0.1% SDS, 2 mM EDTA) with Protease and Phosphatase Inhibitor Cocktail (Thermo Fisher, Waltham, MA USA, USA) added just before use. Protein concentration was determined with a BCA Protein Assay kit (Thermo Fisher Pierce, Waltham, MA USA). Then, 10 µg vagina positive control and 40 µg L1-L2 ATGko protein (3 separate samples) were run on a Novex WedgeWell 4–20% Tris-Glycine Gel (Thermo Fisher Invitrogen, Waltham, MA, USA) and transferred to a PVDF membrane (Merck Millipore, Burlington, MA, USA). MPV.B9 (1:200) and Anti-mouse IgG1 HRP (Thermo Fisher Pierce, Waltham, MA, USA) were used to probe for L1 in Western blot analysis. An IP/Western was also conducted for the tail tissues. Then, 25 µg positive control and 3 separate L1-L2ATGko samples were incubated overnight at 4 °C with 2 µg MPV.A4 mab with end over end mixing. Then, 20 µL protein A/G agarose beads were added for 1 h followed by 4 washes of the pellets with IP buffer. The samples were run and probed as described above. Protein loading controls were confirmed by Coomassie stain (data not shown).

### 2.7. Statistical Analysis

The data were statistically analyzed with one-way ANOVA analysis in Sigmaplot 12 software. A Mann–Whitney rank-sum test and unpaired Student’s *t*-test were used by comparing differences between two groups. Differences were significant at *p* < 0.05.

## 3. Results

### 3.1. Persistent Lesions Were Induced by L1ATGko-2m at Cutaneous Sites

According to an in vitro study, the first two methionines that potentially encode the MmuPV1 L1 protein are not necessary for proper VLP production [21]. To test these findings in vivo, we generated an ATG to ACG mutant of these two methionines (designated as L1ATGko-2m), which would result in an L1 of 508aa (Figure 1B) when compared with the 535aa L1 of the putative wild type (Figure 1A). These mutations did not change the coding sequence of the overlapping L2. The mutant genome was encapsidated in HPV31 L1/L2 capsids to generate quasiviruses. We tested the infectivity of this L1 mutant at two cutaneous sites, the tail and muzzle; tumor growth was monitored, and photographic images were recorded. As shown in Figure 1D, multiple exophytic, verrucous tumors grew at both tail and muzzle sites infected with the mutant QV similar to wild-type infections (Figure 1C). Viral particles were easily visualized in the mutant-induced tumor tissues (Figure 1D). The introduced mutations were retained in the lesions as verified by DNA sequencing (Figure 1D).

### 3.2. The L1ATGko-4m Mutant Induced Persistent Infections at Cutaneous and Mucosal Sites

**Figure 2:**
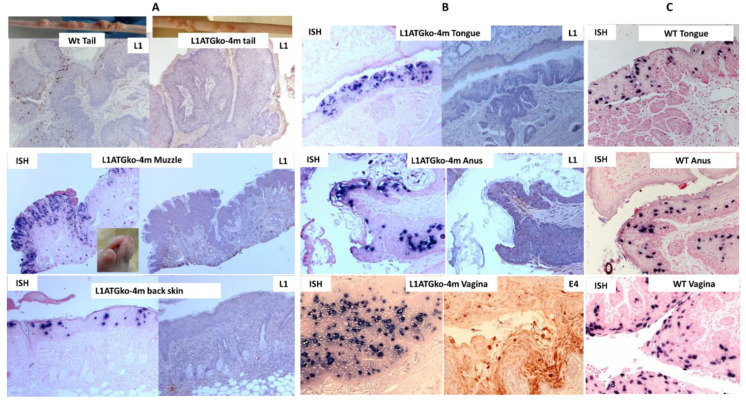
Mouse papillomavirus (MmuPV1) DNA was detected in both cutaneous (**A**) and mucosal (**B**) sites of animals infected with the L1ATGko-4m mutant. Viral capsid protein L1 was strongly positive in the wild-type MmuPV1-induced tail lesion (**A**, left L1, 20×) but not in the L1ATGko-4m mutant-induced tail lesion (**A**, right L1, 20×) by immunohistochemistry (IHC). Full-length L1 was also absent in other lesions including the muzzle, back skin, anus, and tongue initiated by the L1ATGko-4m mutant by MPV.B9 (L1 panel). Viral DNA expression was strongly positive in representative muzzle lesions (**A**, 20×), back skin (**A**, 20×), tongue (**B**, 20×), anal tract (**B**, 20×), and vaginal tract (**B**, 20×) by in situ hybridization (ISH), as shown in wild-type tissues (**C**, 20×). Interestingly, E4 was moderately to strongly cytoplasmic positive within the vaginal epithelium by immunohistochemistry (IHC) using an in-house rabbit polyclonal antibody against the mouse papillomavirus E4 protein (**B**, 20×).

Since mutants with abrogation of the first two methionines displayed an infection pattern similar to that of the wild type, we generated the next L1 mutant with the first four start codons mutated from ATG to ACG and labeled the mutant L1ATGko-4m. The L1ATGko-4m mutant was tested at both cutaneous (Figure 2A) and mucosal (Figure 2B) sites. The cutaneous sites including the tail, muzzle, and back were found to be susceptible to infection by L1ATGko-4m, but the appearance of the cutaneous lesions was delayed, and lesions were smaller than those induced by the wild-type virus (Figure 2A). Viral DNA could be detected throughout the suprabasal epithelial layers in all cutaneous and mucosal sites (tongue, anus, and vagina) by in situ hybridization (ISH) (Figure 2A,B) as shown in wild-type lesions (Figure 2C). L1 expression was absent in the lesions by immunohistochemistry (IHC) using an in-house monoclonal antibody (MPV.B9) against full-length L1, thus confirming the absence of full-length L1 protein in the tissues (Figure 2A,B). We subsequently tested E4 protein expression using a polyclonal antibody. E4 expression could be readily detected, as shown in one example of infected vaginal tissues, indicating that early events in the viral life cycle were intact (Figure 2B). Therefore, L1 absence did not impact MmuPV1 infection negatively.

### 3.3. L2 Is Not Required for Tumor Growth in Cutaneous Tissues of Nude Mice

**Figure 3:**
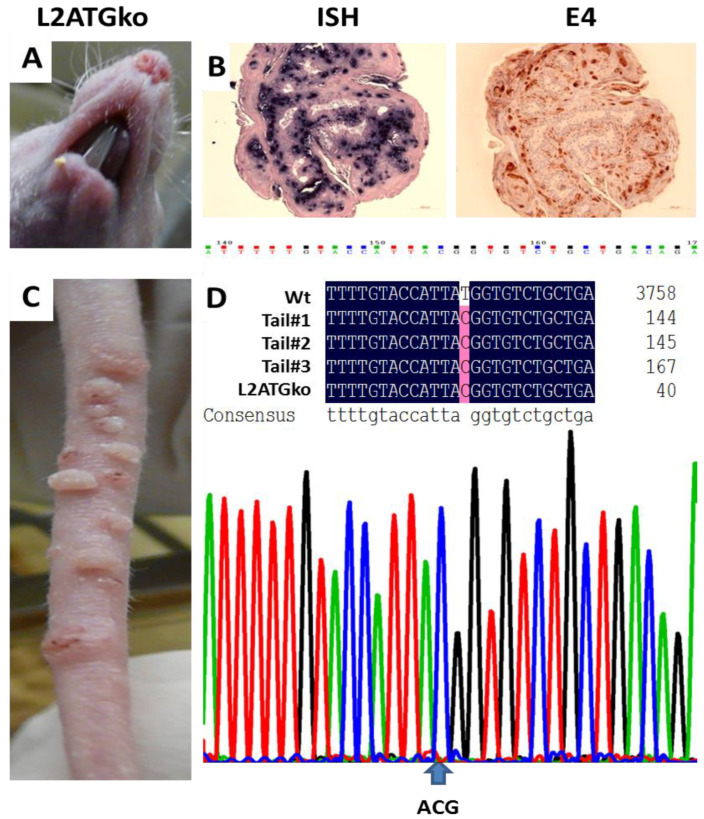
The L2ATGko mutant-induced tumor growth at tails of the nude mice. More tail lesions (**C**) than muzzle lesions (**A**) were detected. Viral DNA was detected in the suprabasilar epidermis of muzzle lesions by in situ hybridization (ISH), and viral E4 protein was detected in the same regions of the muzzle lesions by immunohistochemistry (IHC) (**B**, 20×). DNA extracted from tail lesions induced by the L2 mutant maintained the ATG–ACG mutation. Input L2ATG mutant DNA was aligned with the amplified DNA from lesions (**D**).

The productive stage of the viral life cycle, in which progeny virus is made, arises within the terminally differentiating compartment of stratified squamous epithelia [30,31]. To determine the function of L2 in the MmuPV1 life cycle, we generated the L2ATGko mutant by changing the start codon from ATG to ACG and labeled the mutant L2ATGko. The mutation was confirmed by DNA sequencing (Figure 3D). Initially, we tested infection at two cutaneous sites (muzzle and tail) in two animals (Figure 3A,C). Four weeks after viral infection, lesions were found on one of the tails of these L2 mutant-infected animals. About eight weeks post-infection, lesions appeared on the second tail (Figure 3C). Interestingly, no visible muzzle lesion was found until week twenty-three on one of the animals; later, a small muzzle lesion was found on the second animal as well (Figure 3A). Viral DNA and E4 protein were detected in the upper stratum spinosum and stratum granulosum layers of the epithelium by in situ hybridization and immunohistochemistry respectively (Figure 3B). The L2 ATG mutation in the DNA extracted from the lesions induced by the mutant was confirmed by rolling cycle amplification with high fidelity followed by DNA sequence analysis. As shown in Figure 3D, the mutation was retained in the lesions.

### 3.4. L2 Is Not Required for L1 Expression but Influences Full-Length L1 Location in Infected Cells

**Figure 4:**
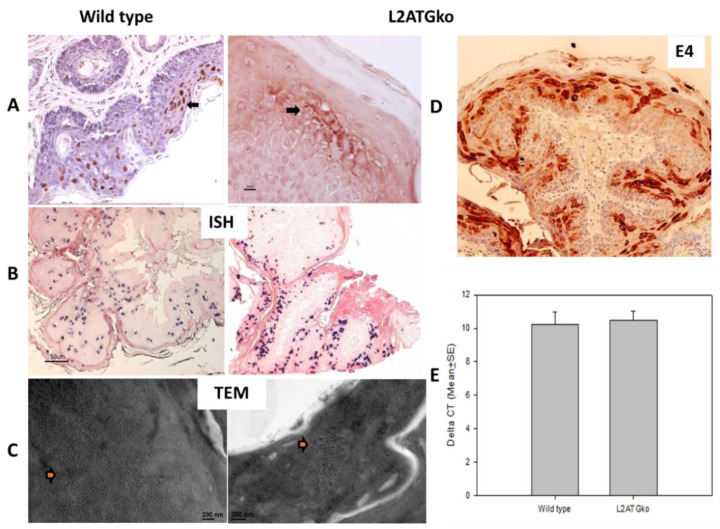
L2 is not required for L1 expression but influences L1 location. L1 expression in L2ATGko mutant lesions was mild–moderate and predominately present in the cytoplasm, while the wild-type lesions demonstrated moderate nuclear expression (**A**, 20×) although a comparative amount of viral DNA was detected (**B**, 10×). In addition, one of the two tail lesions exhibited viral particles by EM (arrows), although detection required exhaustive searching; wild-type lesions contained far more abundant viral particles (**C**). The cytoplasm of suprabasal epidermal cells was strongly positive for viral E4 protein in the L2ATGko mutant-induced lesions (**D**). Biopsies were harvested from lesions induced by the L2ATGko mutant or wild-type MmuPV1 for DNA extraction. No significant difference was found in the DNA copy number from the wild-type lesions and L2ATGko lesions (**E**, *p* > 0.05, unpaired Student’s *t*-test).

Very few studies have been done to unravel the interactions between L1 and L2 in vivo. In this study we wanted to determine whether the absence of L2 influences the expression of L1 at infected sites. For wild-type virus-induced lesions, full-length L1 was found in the nucleus. An in-house monoclonal antibody MPV.B9 was used for detection [27] (Figure 4A). However, L1 was predominately found in the cytoplasm of L2ATGko mutant-infected cells (Figure 4A). L2 absence did not influence E4 protein expression in the cytoplasm of these tissues (Figure 4D). We further examined whether viral particles were produced by L2ATGko infections. By searching extensively, a few viral particles were found in one tail lesion, and none were found in the other lesion (Figure 4C). Interestingly, intranuclear viral DNA, as detected by ISH, was comparable to that seen with wild-type virus (Figure 4B), and the viral DNA level was comparable to those in wild-type lesions by qPCR (Figure 4E, *p* > 0.05 unpaired Student’s *t*-test). This finding suggests that L2 does not play a role in MmuPV1 DNA amplification. However, the presence of only a limited number of viral particles supports the hypothesis that L2 may play a critical role in viral particle assembly and/or transport in vivo.

### 3.5. Simultaneous Mutations of L1 and L2 Do Not Influence Viral DNA Amplification

One of the best-known hallmarks of HPV infection is the induction of DNA synthesis in the suprabasal layers of the epidermis. This reprogramming of suprabasal cells to support DNA synthesis is thought to be required for amplification of the viral DNA that is necessary to produce progeny virus [32]. To determine whether L1 and L2 play a role in DNA amplification, we constructed a genome in which all four of the potential L1 start codons, as well as the L2 start codon, were mutated (L1-L2ATGko).

**Figure 5:**
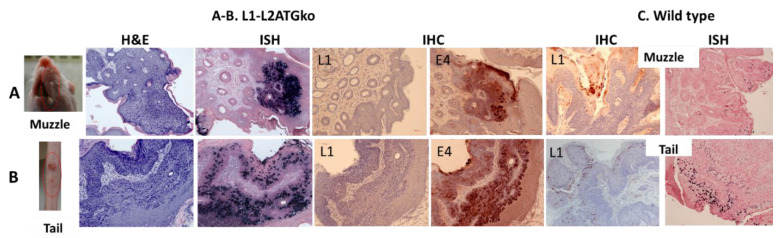
Five animals infected with the L1-L2ATGko developed cutaneous lesions in two repeat experiments. Three of five muzzle sites (Panel **A**) grew visible tumors, and these tumors were multifocally and strongly DNA positive by in situ hybridization (20×, ISH). No L1 staining was found by immunohistochemistry (IHC) with MPV.B9 (20×). E4 expression was intracytoplasmic and marked in these infected tissues (20×). Two of five tails also developed visible lesions (Panel **B**). These lesions were positive for viral DNA and E4 expression but lacked L1 expression (20×). L1 expression was strongly positive in the upper epidermis as detected by IHC using MPV.B9 and ISH from wild-type MmuPV1 infected muzzle and tail tissues (**C**, 20×).

Five HSD nude female mice were infected at both cutaneous (tail and muzzle) and mucosal (tongue, anus, and vagina) sites with the L1-L2ATGko quasiviruses. Out of five animals in repeated experiments, three animals grew tail lesions, and two animals grew muzzle lesions (Figure 5). Compared with the lesions induced by wild-type MmuPV1, these lesions were significantly smaller yet still exhibited the characteristic exophytic, verrucous papillomavirus pathology with a hyperplastic epidermis and variable amounts of acanthosis and hyperkeratosis (Figure 5C). Viral DNA was multifocally present and prominent in the epidermis of these infected tissues as confirmed by in situ hybridization (Figure 5A). Lesions induced by the L1-L2ATGko mutant were further examined for L1 expression using MPV.B9. While there was abundant, moderately intense nuclear expression of L1 detected in wild-type virus-infected tissues (Figure 5B), no full-length L1 expression was found in L1-L2ATGko mutant lesions using MPV B9 antibody (Figure 5A). We subsequently tested E4 protein expression using a polyclonal antibody, and E4 was multifocally and strongly positive in the cytoplasm of the epidermis of all tissues (Figure 5A).

**Figure 6:**
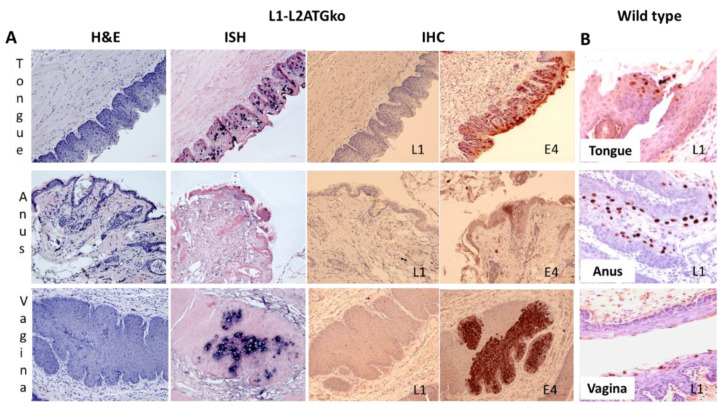
Three mucosal sites (vagina, tongue, and anus) infected with the L1-L2ATGko mutant demonstrated strong viral DNA and E4 expression but absence of L1. Viral DNA positivity was detected in the vaginal tracts and oral cavities of the infected animals of the L1-L2ATGko mutant (**A**, ISH, 20×, and arrows). L1 was undetectable in these tissues by immunohistochemistry (IHC) with MPV.B9 (**A**, 10×). However, all infected tissues showed intracytoplasmic E4 expression ranging from multifocal and light (anus) to more extensive and strongly positive expression (tongue and anus) (**A**). L1 expression is scattered but marked in wild-type MmuPV1 infected tissues by MPV.B9 in the nuclei of infected cells (**B**, 20×).

A similar pattern was observed at the two mucosal sites (vagina and tongue), but very low to absent viral activity was found in the anal tract (Figure 6A). Viral DNA presence in mucosal tissues was confirmed by in situ hybridization (Figure 6A), and no L1 was detected in these tissues when compared with wild-type virus-induced infections (Figure 6B). Viral E4 protein was moderate to strongly positive in the infected vaginal and tongue tissues, indicating the early viral life cycle was not compromised (Figure 6A). In the vaginal tract, unlike the extensive infection along the canal seen in wild-type lesions, only focal segments of virus-positive cells were detected. Interestingly, L1-L2ATGko also targeted the circumvallate papilla (CVP) region of the tongue as seen with the wild-type virus, indicating this tissue tropism was maintained in lesions generated by the mutant genome (Figure 6B).

**Figure 7:**
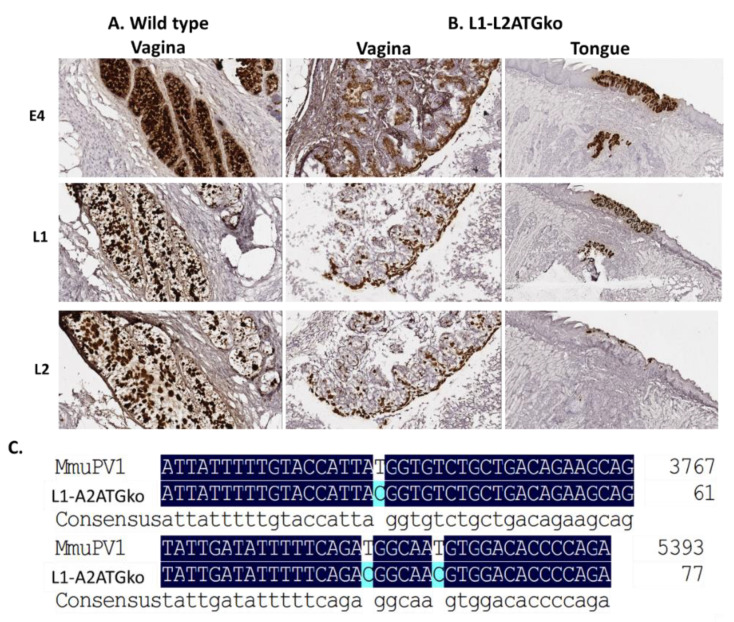
Viral transcripts (E4, L1, and L2) were detected in L1-L2ATGko infected tissues. Both wild-type (**A**) and L1-L2 mutant (**B**) infected tissues were examined for viral transcripts of E4, L1, and L2. All three transcripts (L1, L2, and E4) were found in both wild-type (Vaginal tissue) and L1-L2ATGko mutant (vaginal and tongue tissues). The mutation of L1-L2ATGko mutant was maintained in the lesions and confirmed by DNA sequencing following rolling circle amplification (**C**).

**Figure 8:**
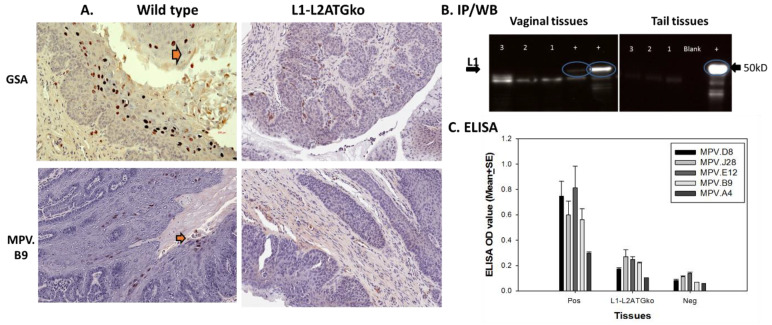
No L1 protein was detected in L1-L2ATGko infected tissues by IHC, ELISA, and IP/Western. Both wild-type and L1–L2 mutant infected tissues were examined for viral L1 using GSA and MPV.B9 antibody. No positive signals were detected in L1-L2ATGko mutant-induced tissues (**A**, right panel), while positive signals could be detected in wild-type infected tissues (left panel, arrows). No L1 positive signals were detected in L1-L2ATGko mutant-infected vaginal tissues by direct Western blot (left, lanes 1–3 from three animals, two wild-type lesions as positive controls “+”). No L1 was detected in L1-L2ATGko tail tissues (right, lanes 1–3 from three animals, one wild-type tail lesion as positive control “+”) in an IP/Western immunoprecipitation using MPV.A4 to pull down L1 and probing the blot with MPV.B9 (**B**). Wild-type and L1-L2ATGko mutant-infected tissues were also tested for L1 by ELISA (**C**) using a panel of in-house monoclonal antibodies including MPV.B9 and MPV.A4 that were raised against the mouse papillomavirus major capsid protein L1. No significantly difference was found between the L1-L2ATGko mutant and the negative group (*p* > 0.05, Mann–Whitney rank-sum test).

To further determine whether viral transcripts were impacted in L1-L2ATGko mutant-induced lesions, we used RNA-ISH to detect L1, L2, and E4 transcripts in these tissues. As shown in Figure 7B, all transcripts were present as shown in wild-type MmuPV1 lesions (Figure 7A) in both vaginal (left panel) and tongue (right panel) tissues by L1-L2ATGko mutant, suggesting that viral transcripts were not abrogated in these infected cells. We failed to detect L1 protein in L1-L2ATGko mutant infected tissues using the MPV.B9 antibody that targets the full-length L1 (Figure 8A). To investigate the possibility of expression of a truncated L1 from the next available methionine (175aa downstream), we used the Group-Specific Antibody (GSA, Dako, USA) of HPV L1 to probe the mutant lesions. GSA is a goat polyclonal antibody that recognizes cross-reactive epitopes located in several sites ranging from amino acids 171 to 475 of papillomavirus L1 [33]. This antibody had been employed successfully with wild-type lesions [20]. No L1 staining was found in the L1-L2ATGko mutant-infected tissues (Figure 8A, right panel), while L1 was readily found in wild-type lesions (Figure 8A, left panel, arrows). We also used MPV.A4, a neutralizing antibody that binds conformational L1 to pull down L1 from both wild-type (10 µg) and three individual L1-L2ATGko (40 µg) samples of tail tissues and detected no L1 signals in L1-L2ATGko mutant infected tail tissues using MPV.B9, a second in house antibody raised against L1 and reported in our previous publications (Figure 8B, right, lanes 1–3 represent three individual samples) [14,25,27]. No L1 signals were detected in L1-L2ATGko infected vaginal tissues (40 µg), as L1 could be seen in two different wild-type controls (10 µg) (Figure 8B, left, lanes 1–3 represent three individual samples) as determined by a direct Western blot. In addition, we tested L1 presence in these tissues using a panel of monoclonal antibodies generated in our laboratory against MmuPV1 L1 by ELISA and found close to background signals by all antibodies tested as seen with MPV.A4 and MPV.B9 (Figure 8C, *p* > 0.05, Mann–Whitney rank-sum test, vs. negative control). Collectively, these observations support the prediction that four ATG to ACG knockouts in L1 completely abrogated L1 expression in these tissues. We do not currently have the tools to investigate whether a truncated L2 could be expressed by the L2ATGko mutant.

### 3.6. Differential Infection Patterns Were Noted in Mucosal Infections with L1 and L2ATGko Mutants

To explore how L1 and L2 influence mucosal infections in the animals, twelve athymic nude mice were divided into four groups (N = 3 mice/group) and each group was infected with wild-type (Table 1), L1ATGko-4m (Table 2), L2ATGko (Table 3), and L1-L2ATGko (Table 4) mutants, respectively, at two mucosal sites (anus and vaginal). Viral DNA was tracked via lavage samples collected over time. Much lower viral DNA copies were detected in both vaginal and anal tracts of the mutant-infected animals when compared with those of wild-type-infected animals. Consistent with what we had found previously (Figure 6), little to no viral DNA was detected in L1-L2ATGko mutant infected anal lavages (Table 4). Interestingly, significant differences were found in viral DNA copy numbers between the vaginal and anal tract in L1ATGko-4m infected mice at all time points post viral infection and in the L1-L2ATGko animals as shown at later time points of wild-type infected animals (*p* < 0.05 unpaired Student’s *t*-test). However, there was no difference in DNA copy number between vagina and anus for the L2ATGko infections (*p* > 0.05 unpaired Student’s *t*-test). These findings indicate that L1 may contribute to the differential tissue susceptibility of the vaginal and anal tracts.

## 4. Discussion

In this study, we characterized the role of the capsid proteins L1 and L2 in the viral life cycle of MmuPV1 using our recently established mouse papillomavirus model, which is a model that displays a broad tissue tropism [14,20]. We generated ATG start codon mutations in L1, L2, as well as both L1 and L2 for the current study and delivered the mutant genomes to the tissues by quasivirus infections [22]. Our data demonstrate that full-length L1 and L2 capsid proteins are not essential for tumor growth at either cutaneous or mucosal sites. The L1ATGko-4m mutant induced lesions at both tail and muzzle sites. The L2ATGko mutant induced more lesions at the tail site than at the muzzle site; however, lesions generated by these mutant genomes were smaller than those generated by the wild type. We postulate that the reduced size could be due to the compromised efficiency of delivery of viral DNA by the quasiviruses compared to wild-type virus. However, the results with L1ATG-2m delivered by HPV31 QV would argue against this. With this mutant, results were similar to those by wild-type virus infection.

The double L1-L2ATGko mutant failed to induce tail lesions in two of the five tested mice, and the lesions were significantly smaller than those induced by L1ATGko or L2ATGko mutants, suggesting that the mutations in this genome influenced the infectivity of the mutants. Full-length L1 expression was abrogated in the L1ATGko-4m and L1-L2ATGko-infected tissues as determined by probing with a panel of anti-L1 antibodies including MPV.A4 and MPV.B9, but viral DNA replication, viral transcripts of E4, L1, and L2, as well as E4 expression were not compromised. No L1 signals in L1-L2ATGko mutant-infected tissues were found using the antibody against group-specific antigen (GSA), which targets the carboxy-terminus of L1 [33]. Interestingly, full-length L1 expression in the lesions induced by the L2 mutant was predominately detected in the *cytoplasm*, suggesting that the absence of L2 has an impact on the location of full-length L1 expression and/or transport in vivo. We postulate that in the absence of L2, the nuclear localization signal at the C terminus of full-length L1 is disactivated, whereas in the presence of L2, the influence of L2 protein predominates.

The L1 and L2 proteins of the cottontail rabbit papillomavirus have been found to be non-essential for tumor outgrowth in the rabbit papillomavirus model [4,6,7,8,9]. Other studies demonstrated that L2 is critical for the infectivity of pseudoviruses [34,35]. In another study, the authors used a raft culture system to study the function of HPV31L2 and demonstrated that L2 was not required for the formation of virus particles but was critical for the stability of the viral genome as well as the production of infectious virus [11]. Our studies reported here are consistent with these findings in that we found very limited numbers of viral particles in lesions induced by the L2 mutant.

DNA replication in lesions induced by the mutant genomes was comparable to that of wild-type lesions. This suggests that neither L1 nor L2 protein is required for viral replication. On the other hand, localization of the full-length L1 protein was dependent upon the expression of L2. For example, the mutation of the L2 ATG resulted in the relocation of full-length L1 expression to the cytoplasm. Our work has been hampered by the lack of an antibody to L2, and studies are ongoing to further elucidate the nature of L1/L2 interactions. The findings presented here support earlier in vitro findings that the likely start codon for L1 is the 3rd methionine at amino acid position 28 [21]. The L1ATG ko-2m genome behaved in a manner similar to wild type in both its infectivity patterns and its ability to support virus particle formation by TEM.

In conclusion, we have demonstrated that both papillomavirus capsid proteins (L1 and L2) are dispensable for viral DNA replication and tumor growth in vivo. However, L1 and L2 do appear to contribute to viral production and differential tissue susceptibility.

## Figures and Tables

**Table 1:** Viral DNA copy numbers in vaginal (V) and anal (A) tracts of three mice infected with wild-type MmuPV1 over time.

Mouse	Week 4	Week 8	Week 12	Week 19	Week 23
#1V	2.9 × 10^6^	3.3 × 10^6^	1.6 × 10^6^	2.98 × 10^6^	3.9 × 10^6^
#2V	2.2 × 10^6^	1.5 × 10^6^	1.8 × 10^6^	3.48 × 10^6^	4.3 × 10^6^
#3V	2.9 × 10^6^	1.1 × 10^6^	9.9 × 10^5^	2.97 × 10^6^	4.2 × 10^6^
#1A	2.7 × 10^6^	6.5 × 10^5^	9.1 × 10^5^	7.4 × 10^5^	2.6 × 10^6^
#2A	2.6 × 10^6^	1.3 × 10^6^	1.1 × 10^6^	1.49 × 10^6^	2.5 × 10^6^
#3A	2.5 × 10^6^	1.3 × 10^6^	1.2 × 10^6^	6.36 × 10^5^	2.7 × 10^6^

V—vagina, A—anus. Significant differences were found in viral DNA copy numbers between the vaginal and anal tract in wild-type-infected mice after week 19 post viral infection (*p* < 0.001, unpaired Student’s *t*-test).

**Table 2:** Viral DNA copy numbers in vaginal (V) and anal (A) tracts of three mice infected with L1ATGko-4m mutant MmuPV1 over time.

Mouse	Week 4	Week 7	Week 9	Week 11
#1V	5.7 × 10^5^	2.1 × 10^6^	6.2 × 10^5^	7.1 × 10^5^
#2V	1.6 × 10^6^	1.3 × 10^6^	1.7 × 10^6^	8.6 × 10^5^
#3V	7.2 × 10^5^	3.4 × 10^5^	1.6 × 10^5^	6.7 × 10^5^
#1A	4.6 × 10^5^	ND	ND	ND
#2A	9.0 × 10^5^	6.1 × 10^5^	7 × 10^2^	7.6 × 10^4^
#3A	5.2 × 10^5^	ND	9.7 × 10^4^	ND

V—vagina, A—anus, ND—not detected. Significant differences were found in viral DNA copy numbers between the vaginal and anal tracts in L1ATGko mutant-infected mice at all time points post viral infection (*p* < 0.05, unpaired Student’s *t*-test).

**Table 3:** Viral DNA copy numbers in vaginal (V) and anal (A) tracts of three mice infected with L2ATGko mutant MmuPV1 over time.

Mouse	Week 4	Week 7	Week 9	Week 11
#1V	4 × 10^5^	1.96 × 10^5^	1.1 × 10^5^	2.1 × 10^5^
#2V	4.6 × 10^5^	1.74 × 10^5^	8.7 × 10^4^	6.1 × 10^5^
#3V	6.6 × 10^5^	ND	6.7 × 10^4^	1.1 × 10^5^
#1A	3.7 × 10^5^	ND	4.2 × 10^5^	2.1 × 10^5^
#2A	4 × 10^5^	2.56 × 10^5^	ND	3.9 × 10^5^
#3A	7.3 × 10^5^	ND	ND	1.6 × 10^5^

V—vagina, A—anus, ND—not detected. No significant differences were found in viral DNA copy numbers between the vaginal and anal tract in L2ATGko mutant-infected mice at all time points post viral infection (*p* > 0.05, unpaired Student’s *t*-test).

**Table 4:** Viral DNA copy numbers in vaginal (V) and anal (A) tracts of three mice infected with L1-L2ATGko mutant MmuPV1 over time.

Mouse	Week 4	Week 7	Week 9	W11
#1V	5 × 10^5^	ND	7.5 × 10^4^	3.5 × 10^5^
#2V	5.2 × 10^5^	8.76 × 10^5^	8.3 × 10^5^	1.5 × 10^6^
#3V	6.6 × 10^5^	ND	6.7 × 10^4^	1.1 × 10^5^
#1A	1 × 10^6^	ND	8.5 × 10^4^	2.4 × 10^5^
#2A	4.0 × 10^5^	ND	ND	ND
#3A	8.8 × 10^5^	ND	ND	ND

V—vagina, A—anus, ND—not detected. Significant differences were found in viral DNA copy numbers between the vaginal and anal tract in L1-L2ATGko mutant-infected mice at all time points post viral infection (*p* < 0.05, unpaired Student’s *t*-test).

## Data Availability

All data generated and analyzed during this study are included in this article.

## Abbreviations

MmuPV1	the mouse papillomavirus
IHC	immunohistochemistry
ISH	in situ hybridization
RNA-ISH	RNA-in situ hybridization
IP	immunoprecipitation
TEM	Transmission electron microscopy
